# Influence of Temperature Effect on the Static and Dynamic Performance of Gas-Lubricated Microbearings

**DOI:** 10.3390/mi11080716

**Published:** 2020-07-24

**Authors:** Liangliang Li, Zhufeng Liu, Chongyu Wang, Yonghui Xie

**Affiliations:** 1 School of Energy and Power Engineering, Xi’an Jiaotong University, Xi’an 710049, China; liliangliang@stu.xjtu.edu.cn (L.L.); echo1215@stu.xjtu.edu.cn (Z.L.); 2 MOE Key Laboratory of Thermo-Fluid Science and Engineering, Xi’an Jiaotong University, Xi’an 710049, China; wangcy16@stu.xjtu.edu.cn

**Keywords:** gas-lubricated microbearings, temperature, static and dynamic characteristics, dynamic limit performance, rarefaction effect

## Abstract

Gas-lubricated microbearings are widely applied in multiple fields due to their advantages of high-speed, low friction level and other features. The operating environment of microbearings is complex, and the difference of temperature has an important influence on their comprehensive performance. In this investigation, FEM (finite element method) is employed to investigate the static, dynamic and limit characteristics of microbearings lubricated by different kinds of gas at different temperatures. The results show that the rise of temperature leads to the decline of equivalent viscosity of gas, which weakens the load capacity of microbearings, and furthermore, affects the operating stability of microbearings. The dynamic performances of microbearings at different temperatures are very different, and the two dynamic limit characteristics are more sensitive to temperature when it changes.

## 1. Introduction

Gas-lubricating microbearings have been widely applied in aerospace [1], precision manufacturing [2], micromachine operation [3] and other fields. There is a large difference in the comprehensive performance in different conditions, and the temperature is one of the most important parameters that affects the performance of microbearings. The operating temperature of gas can reach thousands of centigrade in some extreme situations. On the one hand, high temperature puts forward a specific challenge for the manufacture of gas bearing. On the other hand, the existence of the rarefaction effect [4] and the variation of physical properties of gas film will also cause drastic changes in the comprehensive characteristics of the bearing, thus affecting the operating stability of microbearings. Therefore, the temperature-dependent characteristics of gas bearing deserves particular attention.

Temperature factors affect the performance of gas bearing mainly through two aspects. First, a higher temperature will increase the molecular free path [5], suggesting that the rarefaction effect becomes more obvious at a high temperature. Furthermore, the rise in temperature can bring about the increase in gas viscosity, which is an important factor affecting the load capacity and dynamic performance of gas bearing [6]. Following these two combined effects, gas bearing will show complex static and dynamic characteristics in a high temperature environment.

Due to the existence of the rarefaction effect, there are obvious discrepancies in load capacity and dynamic parameters of gas bearing compared with continuum flow model. Scholars have conducted considerable investigations on the rarefied gas effect [5,7,8,9]. Amongst the early studies, the statistics method represented by direct simulation of Monte Carlo (DSMC) [10,11] was dominant, which can accurately capture the microscopic rules of thermal movement of gas molecules as well as predict the mechanical properties of slider gas bearing. However, with the increasing modeling complexity and computational mesh nodes of the gas bearing model, the computation and time cost of DSMC will also dramatically escalate. It is urgent to find a high-precision approximation method. Among the numerical models such as the first order, 1.5 order, second order and FK model [12,13,14,15] proposed previously, the FK model was the most popular way to deal with the rarefied flow. The FK model was firstly proposed by S. Fukui and R. Kaneko [13], the results of which coincide with those of DSMC in the microcosmic state. Due to its lower calculation cost and higher precision, the FK model had been applied in various cases. In recent years, by using the FK model, some scholars [16,17] have found that the rarefaction effect has a large influence on the load capacity and attitude angle of MEMS bearing compared with those in the continuous model.

Compared with the rarefaction effect, temperature affects the gas viscosity more directly. Different kinds of gas have temperature-dependent physical properties, which directly leads to the change of the dynamic parameters of gas bearing. On the one hand, the performance of bearing at different gas temperatures was studied in detail by some researchers such as in literature [5]; however, the dynamic limit performance was not fully considered in these investigations. On the other hand, a single fluid medium [18,19] was investigated in most studies, and there was a lack of systematic investigation on the temperature effect for gas bearing. Therefore, it was necessary to investigate the different types of gas systematically. The performance of microbearings gas bearing usually had a smaller and more precise geometric structure and the rotating speed of which (greater than 10^5^ rpm in most cases) was usually larger than traditional gas bearing [20]. Therefore, the flow characteristic of traditional gas bearing was simpler than that of microbearings due to its very small Knudsen number. In a more comprehensive research field concerning industrial application, some researchers [19,21,22,23,24,25] investigated the damage characteristic and the dynamic performance of gas bearing at high temperatures and developed the corresponding flow field temperature control strategy. However, their studied objects are mainly aimed at traditional gas bearing, and there were few literatures on the microbearings lubricated by gas. Others [26] conducted profound research on radial injection and axial cooling in gas bearing, and the cooling effect at different speeds was verified by experiments. These studies mainly discussed the heat transfer characters of gas bearing and have little relation to the static and dynamic performance of gas bearing.

Based on the description above, it could be found that the mechanism of the temperature effect on the comprehensive performance of microbearings is complex [27,28,29]. For gas-lubricated microbearings, the temperature effect is more noteworthy due to its precise structure and complex and changeable operation situations. However, most of the previous studies focused on the traditional gas bearing, while there is little research on the temperature effect of gas-lubricated microbearings. Furthermore, the working fluid of gas bearing may differ in different working occasions, and the temperature effect of variable working fluids deserves high concern as well. Considering the two points above, four typical gas working fluids are selected in the paper, their static, dynamic and dynamic limit characteristics are fully investigated considering the rarefaction effect, and the results of the comprehensive performance analysis of gas bearing are obtained under different temperature conditions.

The structure of this paper follows 4 steps as follows: In Section 1, the introduction of gas-lubricated microbearings is illustrated. The investigations of previous scholars are demonstrated to draw out the importance of the temperature effect and rarefaction effect, and the structure of this study is described. In Section 2, the numerical method for the solution of lubricated equations considering the rarefaction effect and temperature effect is established and the structural parameters of the investigated gas bearing is given. In Section 3, the main conclusions are proposed, and the discussion is carried out concerning the static and dynamic characteristics of microbearing. In Section 4, the summary of the main results of this investigation is given and a comparison with previous studies is carried out to demonstrate the value of this study.

## 2. Numerical Method

### 2.1. Treatment of Temperature and Rarefaction Effect

The temperature effect on the performance of microbearings is mainly reflected in the gas viscosity and rarefaction effect. The dynamic viscosity of gas and temperature satisfy the following relationship [25]:

(1) $$\mu =\frac{5m}{15d^{2}}{\left(\frac{RT}{\pi }\right)}^{0.5}$$

where *μ* is the gas dynamic viscosity, *m* is the molecular mass of the gas, *d* is the molecular diameter of the gas, *R* is the gas constant and *T* is the temperature. A conclusion can be obtained that the gas dynamic viscosity *μ* is proportional to *T*^0.5^. For the rarefied gas flow, the characteristic inverse Knudsen number (*D*_0_) describing the degree of rarefaction effect is defined as:

(2) $$D_{0}=\frac{P_{a}c}{\mu \sqrt{2RT}}$$

where *c* is the characteristic gas film thickness of the bearing. The characteristic inverse Knudsen number *D*_0_ is inversely proportional to *T*^0.5^.

**Figure 1:**
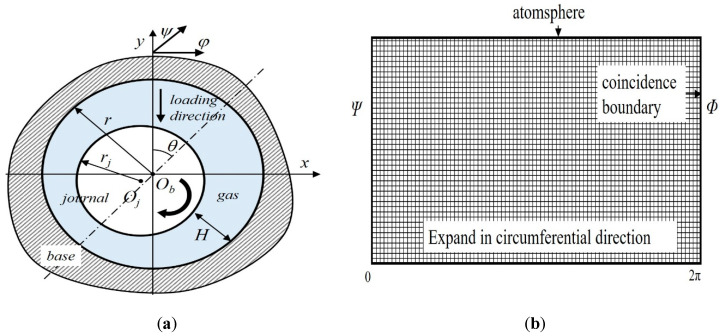
Geometry and mesh of a typical gas-lubricated microbearings (**a**) geometry of bearing; (**b**) mesh of the finite element model (FEM).

Figure 1 shows the geometry and mesh of gas-lubricated microbearings. The mesh number used for the calculation was 2 × 10^4^, which was precise enough to guarantee the convergence of the calculation. The solution was realized by programming using python. Based on the BGK equation of the linear Boltzmann model, S. Fukui and R. Kaneko [13,14] proposed an FK model suitable for any *Kn*. The calculation error between this model and the DSMC method is small, while the calculation time is greatly reduced. In this paper, the FK model was adopted to simulate the rarefaction effect. A fluid control equation considering rarefaction effect was obtained by introducing the FK model into gas lubrication Equation [15]:

(3) $$\frac{\partial }{\partial \varphi }\left[PH^{3}{\bar{Q}}_{p}(D_{0}PH)\frac{\partial P}{\partial \varphi }\right]+\frac{\partial }{\partial \psi }\left[PH^{3}{\bar{Q}}_{p}(D_{0}PH)\frac{\partial P}{\partial \psi }\right]=\Lambda \frac{\partial (PH)}{\partial \varphi }+2\Lambda \frac{\partial (PH)}{\partial \tau }$$

where *P* is the dimensionless pressure, *H* is the dimensionless film thickness, *Λ* is the bearing number, *φ* and *ψ* are the coordinates, ${\bar{Q}}_{p}$ represents the relative dimensionless pressure flow rate, defined as:

(4) $${\bar{Q}}_{p}(D)=-\frac{Q_{P}(D)}{Qcon}$$

where *Q_P_* is the dimensionless pressure flow rate, based on the linear Boltzmann model, *Q_P_* gives

(5) $$Q_{p}=\left\{ \begin{array}{cc} D/6+1.0162+1.0653/D-2.1354/D^{2} & \left(5\leq D\right)\\ 0.13852D+1.25087+0.15633/D-0.00969/D^{2} & \left(0.15\leq D<5\right)\\ -2.22919D+2.10673+0.01653/D-0.0000694/D^{2} & \left(0.01\leq D<0.15\right) \end{array}\right.$$

### 2.2. Control Equations and Solution Method

The fluid domain was discretized by a quadrilateral isoparametric element. By introducing the FK model into the lubrication equation, the calculation model considering the rarefaction effect can be solved using FEM. When gas bearing is in a steady state, the time relative term can be neglected, then Equation (3) can be simplified as:

(6) $$\frac{\partial }{\partial \varphi }\left[PH^{3}{\bar{Q}}_{p}(D_{0}PH)\frac{\partial P}{\partial \varphi }\right]+\frac{\partial }{\partial \psi }\left[PH^{3}{\bar{Q}}_{p}(D_{0}PH)\frac{\partial P}{\partial \psi }\right]=\Lambda \frac{\partial (PH)}{\partial \varphi }$$

The Galerkin method was adopted to transform the lubricated equation and define

(7) $$A_{I}^{e}=\iint_{\Omega^{e}}\left[\frac{H^{3}}{2}\bar{Q}(D)\frac{\partial \Phi }{\partial \varphi }\frac{\partial \Phi^{T}}{\partial \varphi }+\frac{H^{3}}{2}\bar{Q}(D)\frac{\partial \Phi }{\partial \psi }\frac{\partial \Phi^{T}}{\partial \psi }\right]d\varphi d\psi$$

(8) $$B_{I}^{e}=\iint_{\Omega^{e}}-\Lambda H\frac{\partial \Phi }{\partial \varphi }\Phi^{T}d\varphi d\psi$$

Equation (6) can be written as the following discrete form of the finite element:

(9) $$A_{I}^{e}P_{I}^{e}{}^{2}+B_{I}^{e}P_{I}^{e}=0$$

The dimensionless pressure distribution of each discrete node can be gained by solving the non-linear Equation (9) using the Newton–Raphson method. The dimensionless load capacity of the microbearings can be obtained by integrating the dimensionless pressure in two directions as in Equation (10). The dimensionless mass flow, volume flow, friction torque and friction work consumption can be acquired using the numerical integration method as in Equations (11)–(14).

(10) $$\left\{\begin{array}{l} W_{x}\\ W_{y} \end{array}\right\}=\int_{-\frac{L}{2r}}^{\frac{L}{2r}}\int_{0}^{2\pi }\left(P(\varphi ,\psi )-1\right)\left\{\begin{array}{l} \cos(\varphi -\theta )\\ \sin(\varphi -\theta ) \end{array}\right\}d\varphi d\psi$$

(11) $$Q_{m}=-\int_{-\frac{L}{2r}}^{\frac{L}{2r}}\left(\frac{PH^{3}}{2\Lambda }\frac{\partial P}{\partial \varphi }\right)d\psi$$

(12) $$Q_{v}=-\int_{-\frac{L}{2r}}^{\frac{L}{2r}}\left(\frac{H^{3}}{2\Lambda }\frac{\partial P}{\partial \varphi }\right)d\psi$$

(13) $$T_{b}=\int_{-\frac{L}{2r}}^{\frac{L}{2r}}\int_{0}^{2\pi }\left(\frac{\Lambda }{6}\frac{1}{H}-\frac{H}{2}\frac{\partial P}{\partial \varphi }\right)d\varphi d\psi$$

(14) $$N=\int_{-\frac{L}{2r}}^{\frac{L}{2r}}\int_{0}^{2\pi }\left(\frac{H}{2}\frac{\partial P}{\partial \varphi }+\frac{\Lambda }{6}\frac{1}{H}\right)d\varphi d\psi$$

When the gas-lubricated microbearings works in the dynamic case, the small perturbation method can be used to solve Equation (3). Supposing that a small distribution is imposed on the rotor, and the perturbation amplitude is defined as (*E*_0_,*Θ*_0_), the rotor axis coordinates (*ε*,*θ*) can be expressed as the sum of the static position coordinates (*ε*_0_,*θ*_0_) and perturbation position. Similarly, the gas film pressure can be processed using the small perturbation method. Then, substituting perturbation pressure into Equation (3), the partial derivative method can be used [17,30]. The equations in two directions can be decoupled:

(15) $$\begin{array}{l} \frac{\partial }{\partial \varphi }\left[P_{0}H_{0}{}^{3}{\bar{Q}}_{p}(P_{0},H_{0})\frac{\partial P_{d0}}{\partial \varphi }\right]+\frac{\partial }{\partial \psi }\left[P_{0}H_{0}{}^{3}{\bar{Q}}_{p}(P_{0},H_{0})\frac{\partial P_{d0}}{\partial \psi }\right]\\ +\frac{\partial }{\partial \varphi }\left\{P_{d0}H_{0}{}^{3}\left[{\bar{Q}}_{p}(P_{0},H_{0})\frac{\partial P_{0}}{\partial \varphi }+P_{0}\frac{\partial P_{0}}{\partial \varphi }{\frac{\partial {\bar{Q}}_{p}}{\partial P}|}_{\left(P_{0},H_{0}\right)}\right]\right\}\\ +\frac{\partial }{\partial \psi }\left\{P_{d0}H_{0}{}^{3}\left[{\bar{Q}}_{p}(P_{0},H_{0})\frac{\partial P_{0}}{\partial \psi }+P_{0}\frac{\partial P_{0}}{\partial \psi }{\frac{\partial {\bar{Q}}_{p}}{\partial P}|}_{\left(P_{0},H_{0}\right)}\right]\right\}\\ +\frac{\partial }{\partial \varphi }\left\{H_{d0}\left[3P_{0}H_{0}{}^{2}{\bar{Q}}_{p}(P_{0},H_{0})\frac{\partial P_{0}}{\partial \varphi }+P_{0}H_{0}{}^{3}\frac{\partial P_{0}}{\partial \varphi }{\frac{\partial {\bar{Q}}_{p}}{\partial H}|}_{\left(P_{0},H_{0}\right)}\right]\right\}\\ +\frac{\partial }{\partial \psi }\left\{H_{d0}\left[3P_{0}H_{0}{}^{2}{\bar{Q}}_{p}(P_{0},H_{0})\frac{\partial P_{0}}{\partial \psi }+P_{0}H_{0}{}^{3}\frac{\partial P_{0}}{\partial \psi }{\frac{\partial {\bar{Q}}_{p}}{\partial H}|}_{\left(P_{0},H_{0}\right)}\right]\right\}\\ =\Lambda \frac{\partial }{\partial \varphi }(H_{0}P_{d0}+P_{0}H_{d0})+2i\Lambda \Omega (H_{0}P_{d0}+P_{0}H_{d0}) \end{array}$$

(16) $$\begin{array}{l} \frac{\partial }{\partial \varphi }\left[P_{0}H_{0}{}^{3}{\bar{Q}}_{p}(P_{0},H_{0})\frac{\partial P_{E}}{\partial \varphi }\right]+\frac{\partial }{\partial \psi }\left[P_{0}H_{0}{}^{3}{\bar{Q}}_{p}(P_{0},H_{0})\frac{\partial P_{E}}{\partial \psi }\right]\\ +\frac{\partial }{\partial \varphi }\left\{P_{E}H_{0}{}^{3}\left[{\bar{Q}}_{p}(P_{0},H_{0})\frac{\partial P_{0}}{\partial \varphi }+P_{0}\frac{\partial P_{0}}{\partial \varphi }{\frac{\partial {\bar{Q}}_{p}}{\partial P}|}_{\left(P_{0},H_{0}\right)}\right]\right\}\\ +\frac{\partial }{\partial \psi }\left\{P_{E}H_{0}{}^{3}\left[{\bar{Q}}_{p}(P_{0},H_{0})\frac{\partial P_{0}}{\partial \psi }+P_{0}\frac{\partial P_{0}}{\partial \psi }{\frac{\partial {\bar{Q}}_{p}}{\partial P}|}_{\left(P_{0},H_{0}\right)}\right]\right\}\\ +\frac{\partial }{\partial \varphi }\left\{\cos(\varphi -\theta )\left[3P_{0}H_{0}{}^{2}{\bar{Q}}_{p}(P_{0},H_{0})\frac{\partial P_{0}}{\partial \varphi }+P_{0}H_{0}{}^{3}\frac{\partial P_{0}}{\partial \varphi }{\frac{\partial {\bar{Q}}_{p}}{\partial H}|}_{\left(P_{0},H_{0}\right)}\right]\right\}\\ +\frac{\partial }{\partial \psi }\left\{\cos(\varphi -\theta )\left[3P_{0}H_{0}{}^{2}{\bar{Q}}_{p}(P_{0},H_{0})\frac{\partial P_{0}}{\partial \psi }+P_{0}H_{0}{}^{3}\frac{\partial P_{0}}{\partial \psi }{\frac{\partial {\bar{Q}}_{p}}{\partial H}|}_{\left(P_{0},H_{0}\right)}\right]\right\}\\ =\Lambda \frac{\partial }{\partial \varphi }(H_{0}P_{E}+P_{0}H_{E})+2i\Lambda \Omega (H_{0}P_{E}+P_{0}H_{E}) \end{array}$$

where *P_E_* and *P_Θ_* are the derivatives of *P*_*d*0_ to *E*_0_ and *Θ*_0_, *H_E_* and *H_Θ_* are the derivatives of *H*_*d*0_ to *E*_0_ and *Θ*_0_. The derivative of the dimensionless flow rate gives

(17) $$\frac{\partial {\bar{Q}}_{p}}{\partial P}=\frac{\partial {\bar{Q}}_{p}}{\partial D}\frac{\partial D}{\partial P}=D_{0}H\frac{\partial {\bar{Q}}_{p}}{\partial D}$$

(18) $$\frac{\partial {\bar{Q}}_{p}}{\partial H}=\frac{\partial {\bar{Q}}_{p}}{\partial D}\frac{\partial D}{\partial H}=D_{0}P\frac{\partial {\bar{Q}}_{p}}{\partial D}$$

Furthermore, the Galerkin method was used to obtain the finite element form of these two equations. By solving the large coefficients matrix equations, the numerical distribution of the partial derivatives *P_E_* and *P_Θ_* in the solution domain can be obtained in the two directions. The dimensionless damping and stiffness of microbearings can be gained according to Equations (19)–(22):

(19) $$-\int_{-\frac{L}{2r}}^{\frac{L}{2r}}\int_{0}^{2\pi }P_{E}\cos(\varphi -\theta )d\varphi d\psi =K_{y\varepsilon }+i\Omega D_{y\varepsilon }$$

(20) $$-\int_{-\frac{L}{2r}}^{\frac{L}{2r}}\int_{0}^{2\pi }P_{E}\sin(\varphi -\theta )d\varphi d\psi =K_{x\varepsilon }+i\Omega D_{x\varepsilon }$$

(21) $$-\int_{-\frac{L}{2r}}^{\frac{L}{2r}}\int_{0}^{2\pi }P_{\Theta }\cos(\varphi -\theta )d\varphi d\psi =K_{y\theta }+i\Omega D_{y\theta }$$

(22) $$-\int_{-\frac{L}{2r}}^{\frac{L}{2r}}\int_{0}^{2\pi }P_{\Theta }\sin(\varphi -\theta )d\varphi d\psi =K_{x\theta }+i\Omega D_{x\theta }$$

Converting the solution parameters to the standard coordinate system, 8 dynamic coefficients under different working conditions can be obtained.

### 2.3. Model Parameters and Boundary Condition

The structural and operation parameters of gas-lubricated microbearings studied in this paper are shown in Table 1. The static and dynamic performance of four kinds of representative gas were investigated in this paper at different temperatures. Due to the high speed of the microbearings, the operating eccentricity ratio was greater than 0.8, generally.

**Figure 2:**
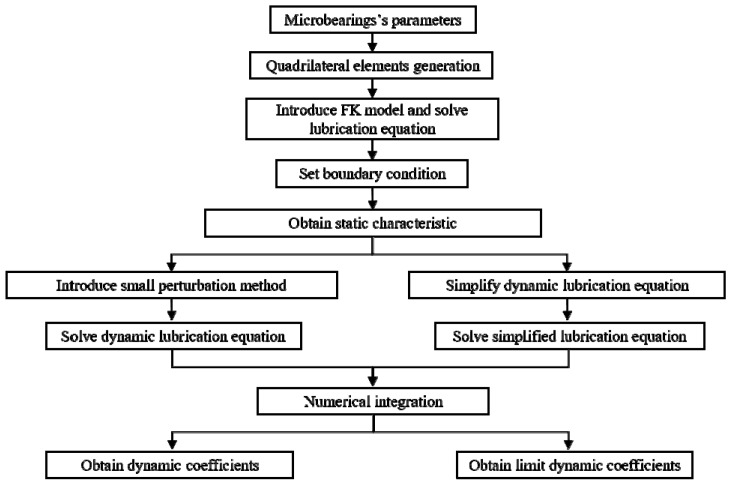
Flow chart of solution.

Figure 2 shows the flow chart of solution and the solution process can be described as: The numerical model calculation domain was constructed firstly according to the parameters of the microbearings in Table 1, then the calculation domain was discretized into quadrilateral elements and all the nodes and elements information of the mesh were obtained. For the static calculation, the coefficients matrix was assembled by FEM [31]. The periodic symmetry boundary condition and the Dirichlet boundary condition were applied to the corresponding boundary. The Newton–Raphson method was applied to gain the numerical nonlinear solution of equations iteratively and then the distribution of the dimensionless pressure distribution was obtained. Numerical integration was adopted to acquire various static parameters. Based on the static results, the dynamic characteristics of microbearings could be obtained using the small perturbation method. Then the PDE (partial differential equation) was solved and the distribution of each partial derivative in the solution domain was obtained. Furthermore, numerical integration and coordinate system transformation were adopted to obtain the dynamic characteristic coefficients of the bearing in the standard solution coordinate system. The configuration of the workstation used for calculation was Intel(R) Core(TM) i5-9400f CPU@2.90GHz, RAM 32G, and the cumulative calculation time was 54 h.

### 2.4. Example Case

To illustrate the solution method and rarefaction effect of the calculation model, an example case is discussed in this section, and the systematic discussion of the temperature effect is carried out in Section 3. In this section, the air was chosen as the working fluid, the comparison between the continuous model and rarefaction model was made for the microbearings (*ε* in the range of 0.8~0.9, *L*/*r* = 0.1, *r* = 2 mm, *n* = 500,000 rpm).

**Figure 3:**
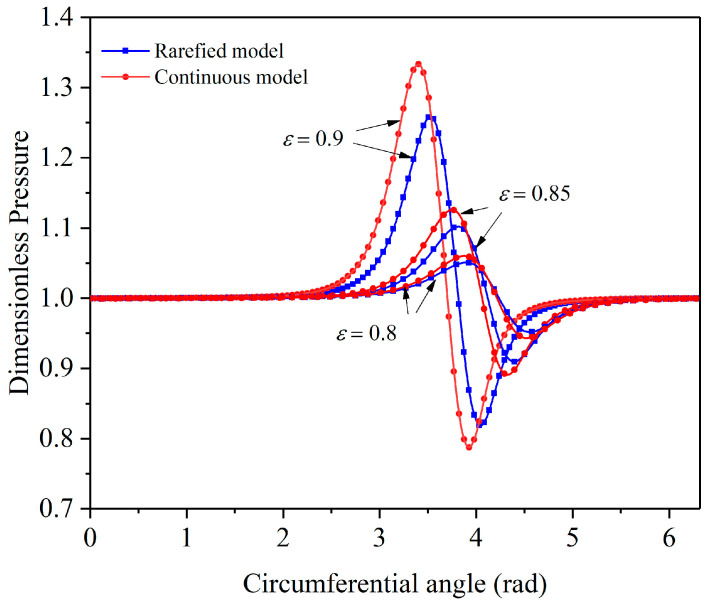
Center pressure comparison of two flow models.

Figure 3 shows the circumferential pressure curve of a micro gas bearing under the condition of the rarefied flow and continuous flow model. Under different eccentricity ratios, the maximum dimensionless pressure of the rarefied flow model decreased compared with the continuous flow model, and the position of the maximum pressure moved in the positive circumferential direction, indicating that the influence of the rarefaction effect on the bearing performance was not negligible for the gas-lubricated microbearings.

**Figure 4:**
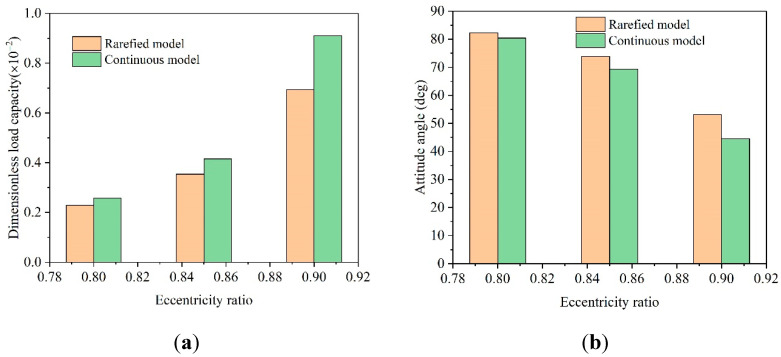
Static performances comparison between two calculation models (**a**) variation of dimensionless load capacity with eccentricity ratio; (**b**) variation of attitude angle with eccentricity ratio.

Figure 4 shows the load capacity and attitude angle under three different eccentricities solved by the continuous flow and FK model, respectively. For high-speed gas bearings, when the rated eccentricity ratio of the bearing changed from 0.8 to 0.9, load capacity increased rapidly, the attitude angle decreased and the influence of the rarefaction effect became increasingly obvious. When considering the rarefaction effect, the load capacity of the bearing was reduced, and the attitude angle increased slightly, which implied that the stability of the microbearings was weakened.

## 3. Results and Discussion

### 3.1. Effect of Temperature on Static Performance

**Figure 5:**
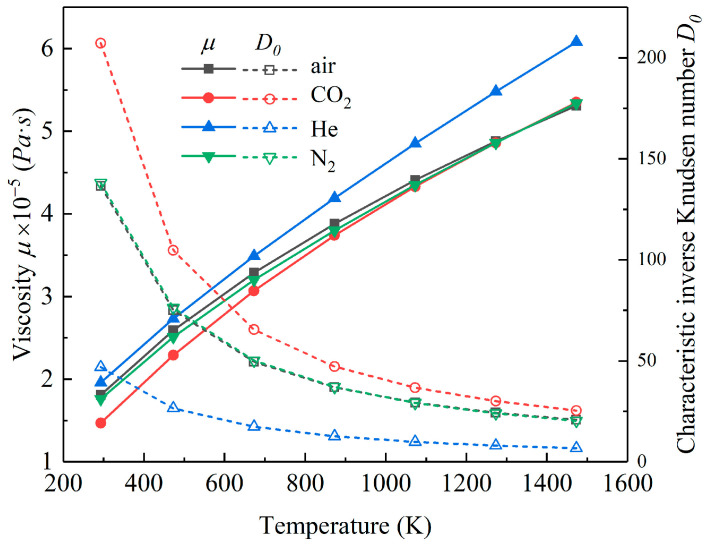
Variation of *μ* and *D*_0_ at different temperatures.

Figure 5 shows the variation of the dynamic viscosity and characteristic inverse Knudsen number of the four kinds of typical gas studied in this paper at different temperatures.

For the four types of gas investigated in this paper, as the proportion of N_2_ in the air was 78%, the physical properties and inverse Knudsen number of air and N_2_ were similar in the rarefied state. The viscosity of He was low due to its small molecular mass and molecular diameter, which led to the largest *Kn* in the rarefied state. In summary, the rarefaction effect of He was the most obvious, while CO_2_ was opposite to He because of its largest molecular mass and molecular diameter.

**Figure 6:**
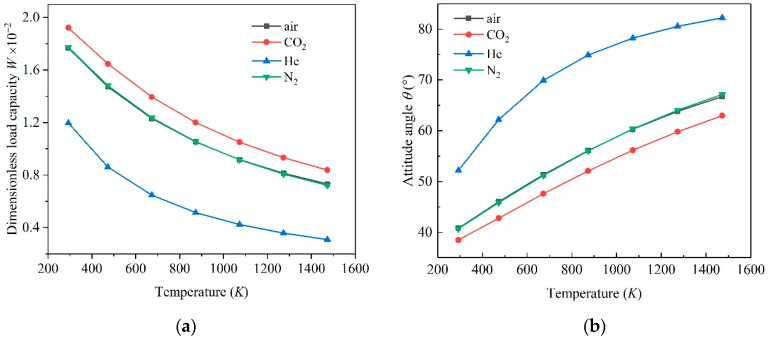
Influence of temperature on load capacity (**a**) and attitude angle (**b**).

Figure 6 shows the influence of temperature on the load capacity and attitude angle of microbearings lubricated by four kinds of gas. In general, when the temperature increased, the load capacity showed a decreasing trend at different temperatures. The load capacity of microbearings lubricated by CO_2_ was the largest, while by He was the smallest. The variation of load capacity for gas bearing lubricated by N_2_ and the air were similar in all temperature cases. For the attitude angle, as the temperature increased, the attitude angle of gas bearing showed an increasing trend, especially for gas bearing lubricated by He (more than 80° at 1473K), which meant the microbearings worked under the condition of low stability. For CO_2_ bearings, the attitude angle was the smallest at each temperature, while the attitude angle of air and N_2_ bearings were always similar in value and change trend.

The dynamic viscosity (*μ*) of the gas increased with increasing temperature, which meant an increase in load capacity. In addition, the inverse Knudsen number (*D*) decreased with the temperature increase, which meant that the rarefaction effect became more obvious with temperature increases, and the influence of the rarefaction effect was more intense. The combined influence of these two effects resulted in a decline tendency in the load capacity and an ascend tendency in the attitude angle of the gas-lubricated microbearings. From a macro perspective, an increase in temperature means a decline in equivalent viscosity of gas, which causes a corresponding change in static characteristics.

From the perspective of the physical properties of gas, since the proportion of N_2_ in air was the highest, its equivalent molecular mass and macroscopic equivalent physical properties were close to N_2_. This special feature of N_2_ brought about similar changes in the load capacity and attitude angles of the two cases. For CO_2_, due to its largest relative molecular mass and molecular diameter, the viscosity was greater than other gases, which implied that its load capacity was greater than other gases, while He was just the opposite.

**Figure 7:**
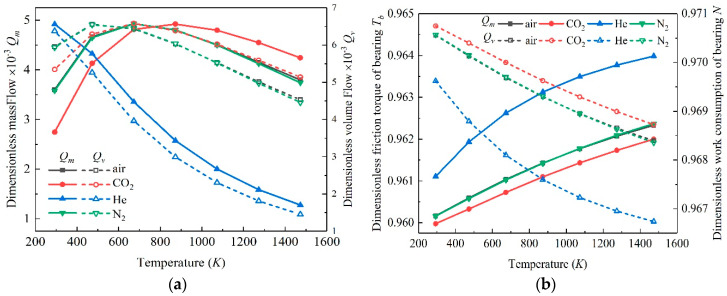
Influence of temperature on flow (**a**) and friction characteristics (**b**).

Figure 7 shows the influence of temperature on gas flow, friction torque and friction work consumption of microbearings lubricated by four kinds of gas. For air, N_2_ and CO_2_, as the temperature increased, the dimensionless mass flow rate and volume flow rate of showed an increasing trend and then decreased, while for He, the dimensionless mass flow rate and volume flow rate showed a decreasing trend. For the dimensionless friction torque of the microbearings inner surface, as the temperature rose, it showed an increasing trend, and the dimensionless friction torque of He was the largest, while the dimensionless friction torque of the CO_2_ was the smallest. The dimensionless friction work consumption was the opposite. In the four cases, the dimensionless friction work consumption showed a downward trend, and the friction work consumption of CO_2_ was the largest.

### 3.2. Effect of Temperature on the Dynamic Characteristics of Microbearings

**Figure 8:**
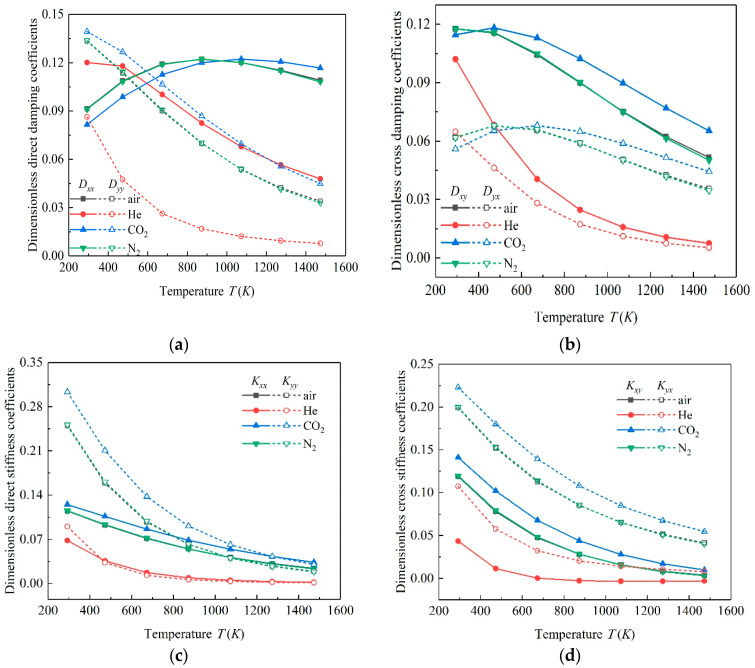
Variation of dynamic coefficients. (**a**) direct damping coefficients; (**b**) cross damping coefficients; (**c**) direct stiffness coefficients; (**d**) cross stiffness coefficients.

Based on the static solution of microbearings, the small perturbation method was adopted to solve the dynamic problem. The dynamic characteristics of gas bearing lubricated by multiple working mediums under different temperatures were obtained. Figure 8 gives the variation rules of eight coefficients of all investigation cases. It can be found that the dynamic performance of the microbearings under different temperatures were quite different, indicating that the temperature affected the dynamic performance of the microbearings distinctly.

When the temperature range was between 293 and 1473 K, for air, N_2_ and CO_2_, *D_xx_* increased first and then decreased with the increase in temperature, while for gas bearing lubricated by He, *D_xx_* decreased. *D_yy_* decreased in all the cases with the increase in temperature. *D_xy_* decreased for all the cases generally, but for CO_2_ gas bearing, *D_xy_* increased slightly when temperature changed from 293 to 473 K. *D_yx_* decreased for microbearings lubricated by He, while the other three showed a slightly upward trend in the temperature range of 293 to 500 K, and then decreased. As for the dimensionless stiffness coefficients, the values of *K_xx_*, *K_yy_*, *K_xy_* and *K_yx_* showed a downward trend in all cases, and their values were positively related to the molecular mass of the gas. That is, the dimensionless stiffness coefficients of CO_2_ gas bearing were the largest, while those of He were smaller in general.

As the physical properties of N2 and air were similar, the dynamic performance of these two kinds of gas showed a similar change regular. The decrease in stiffness meant the capacity of microbearings decreased, which implied that with the ascending temperature, microbearings could not operate normally in the given loading case. The change of damping meant the stability of gas-lubricated microbearings changed correspondingly. When the gas bearing operated in a very high temperature, the damping of bearing decreased obviously, which indicated that the stability decreased. That is why, when the bearing operates in a high temperature condition, the stability should be paid much more attention for the normal working of gas bearing.

### 3.3. Effect of Temperature on +∞ Dynamic Limit Characteristics of Microbearings

The dynamic limit characteristics of gas-lubricated microbearings were an important part for the investigation of the stability of the bearing and dynamic characteristics. In this section, the partial derivative method was used to solve bearing′s +∞ dynamic limit characteristic, and the dynamic characteristic of microbearings was obtained in the case that the perturbation frequency ratio (*Ω*) approached to +∞.

**Figure 9:**
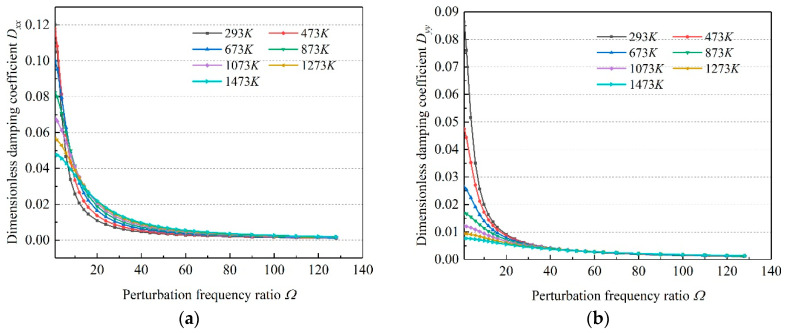

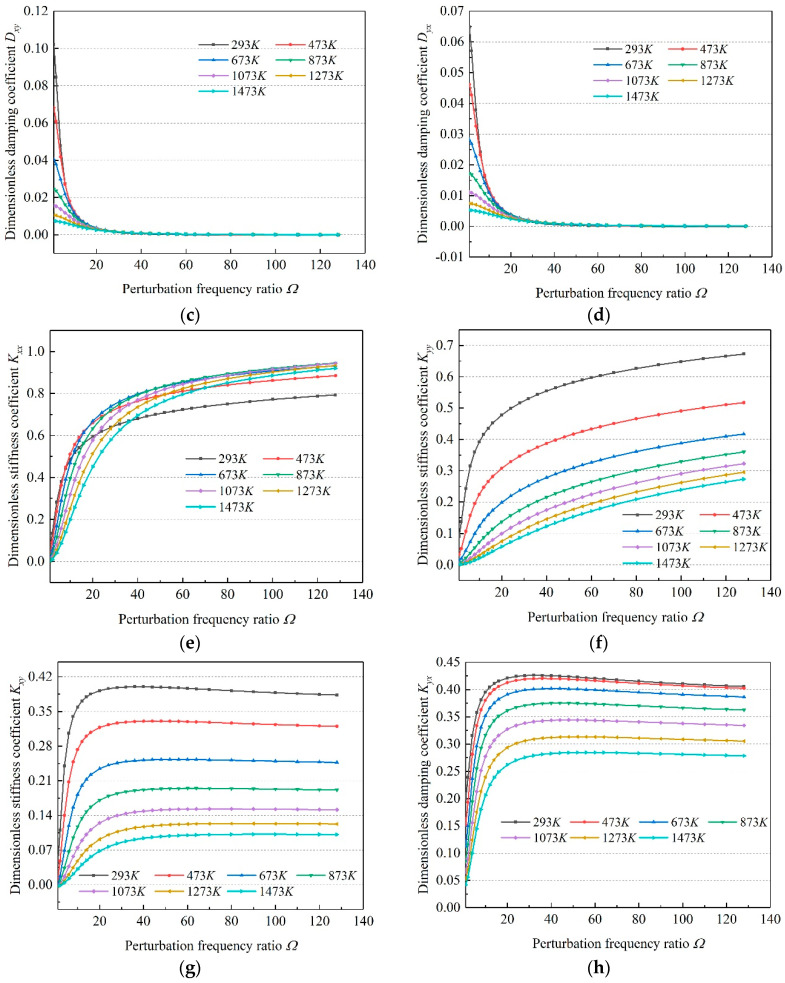
Influence of temperature on the +∞ dynamic limit coefficients. (**a**) *D_xx_*; (**b**) *D_yy_*; (**c**) *D_xy_*; (**d**) *D_yx_*; (**e**) *K_xx_*; (**f**) *K_yy_*; (**g**) *K_xy_*; (**h**) *K_yx_*.

The change curves of eight dynamic coefficients of microbearings lubricated by air when *Ω* changed from 1 to 128 at different temperatures are shown in Figure 9. It can be found that when *Ω* increased, the dimensionless stiffness coefficients and damping coefficients of gas bearing gradually approached a fixed value, respectively. Four damping coefficients: *D_xx_*, *D_yy_*, *D_xy_* and *D_yx_*, tended to zero, which were independent of gas temperature. As for dimensionless stiffness coefficients, the +∞ limit values were different at different temperature conditions. For *K_xx_*, when the temperature was 293 K, the +∞ limit value reached the minimum, and when the temperature was 873 K, the +∞ limit value reached the maximum. *K_yy_*, *K_xy_* and *K_xy_* reached the maximum value when the temperature was 293 K, and the minimum value when the temperature was 1473 K.

For the four types of gas investigated in this paper, the +∞ limit values of the damping coefficients tended to zero, which meant that when the perturbation frequency was large enough, the bearing reached the point where it could not work normally and lost stability completely. In the real case, the perturbation frequency is a certain value, and many methods can be employed to avoid the existence of instability. The +∞ limit values of the stiffness coefficients tended to a certain value, which meant that when the perturbation frequency was large enough, the load capacity of bearing tended to be stable and the value was not related to the change of perturbation. The conclusion of this paper is similar to that of literature [17], but the investigated perturbation frequency range is more widely, which is more accurate.

Table 2 shows the +∞ limit values of the dynamic coefficients of microbearings lubricated by He, CO_2_ and N_2_, respectively. Similar to air, the +∞ limit damping coefficients for three kinds of gas gradually reached the zero line, while *K_xx_*, *K_yy_*, *K_xy_* and *K_yx_* tended to a fixed value, respectively, and the values were different at different temperatures.

### 3.4. Effect of Temperature on Zero Dynamic Limit Characteristics of Microbearings

As *Ω* approaches zero, eight coefficients can be acquired according to the numerical integration method. Similarly, the specific values of eight dynamic coefficients can be obtained.

**Figure 10:**
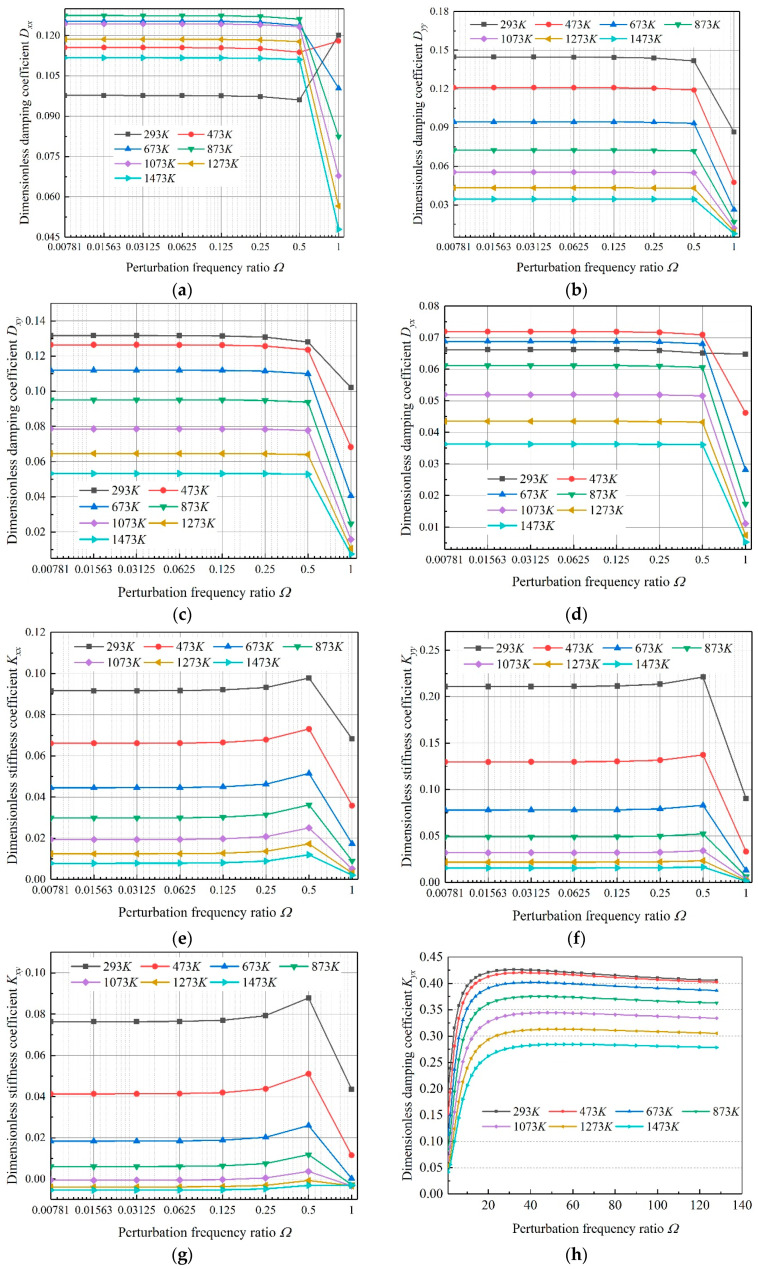
Influence of temperature on the 0 dynamic limit coefficients. (**a**) *D_xx_*; (**b**) *D_yy_*; (**c**) *D_xy_*; (**d**) *D_yx_*; (**e**) *K_xx_*; (**f**) *K_yy_*; (**g**) *K_xy_*; (**h**) *K_yx_*.

Figure 10 shows the change curves of eight dynamic coefficients of microbearings lubricated by air when *Ω* from 2^−7^ to 2^0^ at different temperatures. Eight coefficients of the microbearings tended to a fixed value, respectively, as *Ω* approached zero, and they tended to be different at different temperatures. For *D_yy_|_Ω_*_→0_ and *D_yx_|_Ω_*_→0_, when the temperature was 293 K, the corresponding coefficients values reached the maximum, respectively, and when the temperature was 1473 K, the corresponding coefficients values reached the minimum, which indicated that *D_yy_|_Ω_*_→0_ and *D_yx_|_Ω_*_→0_ decreased with the increase in temperature. For *D_xx_|_Ω_*_→0_, the coefficient value increased with the ascending of temperature. When the temperature was 293 K, *D_xx_|_Ω_*_→0_ reached the minimum, and when the temperature was 1473 K, *D_xx_|_Ω_*_→0_ reached the maximum. For *D_xy_|_Ω_*_→0_, when the temperature was 473 K, the coefficient value reached the maximum value, and when the temperature was 1473 K, the coefficient value reached the minimum value. For the four dimensionless stiffness coefficients, *D_yy_|_Ω_*_→0_, *D_yx_|_Ω_*_→0_, *D_yy_|_Ω_*_→0_ and *D_yx_|_Ω_*_→0_, the maximum value was obtained at 293 K, and the minimum value was obtained at 1473 K, indicating that with the increase in temperature, the four dimensionless stiffness coefficients showed a downward trend.

Similarly, the zero limit values of the dynamic coefficients tended to a certain value corresponding to different cases, which meant that when the perturbation frequency was small enough, the bearing reached the point where it worked in an ideal case. The biggest feature of this case was that there was no disturbance, and the stability of bearing only depended on the nature of bearing structure and operating parameters. The change laws of the zero limit values of the dynamic coefficients agree with those in literature [30], while the results of this paper are suitable for many more types of gas and temperature cases.

Table 3 shows the zero limit values of the dynamic coefficients of microbearings lubricated by He, CO_2_ and N_2_, respectively. Similar to air, the damping and stiffness coefficients for the four kinds of gas tended to a fixed value, respectively, and the values were different at different temperatures.

## 4. Conclusions

Based on the finite element method, a systematic investigation is carried out on the influence of temperature on the comprehensive performance of microbearings in this paper. The lubrication equation is derived considering the rarefaction effect using the FK model, and the finite element solution of the static, dynamic and limit characteristics of gas-lubricated microbearings are obtained.

In summary, gas temperature has a great influence on the static characteristics of microbearings. With the increase in temperature, the loading capacity of four kinds of gas decreases, the attitude angle increases and the gas flow characteristics of the end face vary with each other, the friction torque of the bearing surface increases with the increase in temperature, and the friction work consumption of the bearing decreases. For the dynamic performance, the eight dynamic coefficients of microbearings are greatly affected by temperature, and the change rule of He is quite different from other gas types. For the limit performance, as *Ω* gradually approaches +∞, the damping coefficients *D_xx_*, *D_yy_*, *D_xy_*, *D_yx_* tend to zero and the stiffness coefficients *K_xx_*, *K_yy_*, *K_xy_*, *K_yx_* tend to a certain value, respectively. As *Ω* gradually approaches zero, the eight dynamic limit coefficients of the microbearings tend to a certain value, respectively, and the values are different at different temperatures.

The change of gas temperature will affect the performance of gas bearing from two aspects. On the one hand, the increase in temperature will lead to the increase in gas viscosity, which will increase the load capacity of bearing. On the other hand, the increase in gas temperature will lead to the increase in the rarefaction effect, thus reducing the load capacity of bearing. Under the influence of these two aspects, the equivalent viscosity of gas bearing is the superposition of two factors. In general, the equivalent viscosity of gas decreases with the increase in temperature, which leads to the corresponding changes of static and dynamic properties. 

Generally, the gas with higher molecular mass should be chosen for the occasion with higher requirements of load capacity, while the inert gas (such as He) should be chosen for the occasion with possible corrosion. Moreover, due to the convenience and low price of air, it has a high cost performance. Therefore, in the situation of low requirements, air is a good choice.

## Figures and Tables

**Table 1:** Model parameters of the gas-lubricated microbearings.

Parameters of Microbearings	Values
Length/radius (*L*/*r*)	0.125
Clearance (*c*), μm	10
Rotating speed (*n*), r/min	500,000
Gas type	Air, He, CO_2_, N_2_
Gas temperature (*T_a_*), K	293, 473, 673, 873, 1073, 1273,1473
Viscosity (*η*), Pa·s	correspond to temperature and gas type
Eccentricity ratio (*ε*)	0.9

**Table 2:** +∞ dynamic limit coefficients of different kinds of gas.

Gas Type	Temperature	*K_xx_*|*_Ω_*_→__∞_	*K_yy_|_Ω_*_→__∞_	*K_xy_|_Ω_*_→__∞_	*K_yx_|_Ω_*_→__∞_	*D_xx_|_Ω_*_→__∞_	*D_yy_|_Ω_*_→__∞_	*D_xy_|_Ω_*_→__∞_	*D_yx_|_Ω_*_→__∞_
He	293 K	0.9769	0.8575	0.3783	0.3783	0	0	0	0
	473 K	1.0844	0.7183	0.3181	0.3181	0	0	0	0
	673 K	1.1514	0.6337	0.2474	0.2474	0	0	0	0
	873 K	1.1847	0.5916	0.1928	0.1928	0	0	0	0
	1073 K	1.2019	0.5695	0.1532	0.1532	0	0	0	0
	1273 K	1.2114	0.5571	0.1244	0.1244	0	0	0	0
	1473 K	1.2168	0.5497	0.1032	0.1032	0	0	0	0
CO_2_	293 K	0.8152	1.0912	0.3949	0.3949	0	0	0	0
	473 K	0.8656	1.0135	0.3983	0.3983	0	0	0	0
	673 K	0.9234	0.9305	0.3927	0.3927	0	0	0	0
	873 K	0.9758	0.8591	0.3787	0.3787	0	0	0	0
	1073 K	1.0218	0.7985	0.3589	0.3589	0	0	0	0
	1273 K	1.0605	0.7486	0.3358	0.3358	0	0	0	0
	1473 K	1.0920	0.7086	0.3117	0.3117	0	0	0	0
N_2_	293 K	0.8411	1.0505	0.3976	0.3976	0	0	0	0
	473 K	0.9024	0.9602	0.3959	0.3959	0	0	0	0
	673 K	0.9656	0.8728	0.3821	0.3821	0	0	0	0
	873 K	1.0203	0.8005	0.3597	0.3597	0	0	0	0
	1073 K	1.0662	0.7414	0.3319	0.3319	0	0	0	0
	1273 K	1.1020	0.6959	0.3028	0.3028	0	0	0	0
	1473 K	1.1294	0.6614	0.2749	0.2749	0	0	0	0

**Table 3:** Zero dynamic limit coefficients of different kinds of gas.

	Temperature	*K_xx_*|*_Ω_*_→0_	*K_yy_|_Ω_*_→0_	*K_xy_*|_*Ω*→0_	*K_yx_*|_*Ω*→0_	*D_xx_|_Ω_*_→0_	*D_yy_|_Ω_*_→0_	*D_xy_|_Ω_*_→0_	*D_yx_|_Ω_*_→0_
He	293 K	0.0416	0.0718	0.0157	0.0931	0.1262	0.0904	0.1092	0.0678
	473 K	0.0154	0.0261	−0.0026	0.0500	0.1216	0.0486	0.0710	0.0475
	673 K	0.0041	0.0104	−0.0061	0.0288	0.1022	0.0266	0.0414	0.0285
	873 K	0.0006	0.0050	−0.0058	0.0188	0.0833	0.0169	0.0250	0.0174
	1073 K	−0.0005	0.0027	−0.0049	0.0133	0.0683	0.0122	0.0160	0.0112
	1273 K	−0.0007	0.0016	−0.0042	0.0100	0.0568	0.0094	0.0107	0.0075
	1473 K	−0.0007	0.0010	−0.0036	0.0078	0.0481	0.0077	0.0075	0.0053
CO_2_	293 K	0.1043	0.2603	0.0970	0.2073	0.0873	0.1524	0.1299	0.0600
	473 K	0.0814	0.1754	0.0612	0.1628	0.1055	0.1363	0.1311	0.0697
	673 K	0.0591	0.1115	0.0333	0.1228	0.1194	0.1131	0.1228	0.0718
	873 K	0.0420	0.0726	0.0160	0.0937	0.1261	0.0909	0.1096	0.0679
	1073 K	0.0296	0.0486	0.0059	0.0732	0.1274	0.0722	0.0949	0.0610
	1273 K	0.0205	0.0336	0.0000	0.0583	0.1249	0.0573	0.0806	0.0531
	1473 K	0.0139	0.0239	−0.0033	0.0474	0.1202	0.0460	0.0679	0.0455
N_2_	293 K	0.0920	0.2123	0.0770	0.1831	0.0974	0.1449	0.1317	0.0660
	473 K	0.0668	0.1317	0.0422	0.1363	0.1151	0.1218	0.1267	0.0719
	673 K	0.0451	0.0791	0.0189	0.0989	0.1252	0.0952	0.1125	0.0690
	873 K	0.0300	0.0493	0.0062	0.0738	0.1275	0.0728	0.0954	0.0613
	1073 K	0.0193	0.0317	−0.0007	0.0563	0.1243	0.0552	0.0783	0.0518
	1273 K	0.0120	0.0212	−0.0040	0.0441	0.1181	0.0425	0.0636	0.0429
	1473 K	0.0073	0.0147	−0.0056	0.0354	0.1106	0.0334	0.0516	0.0352

## Nomenclature

*c*	radius clearance/mm
*d*	molecular diameter of the gas
*D*	inverse Knudsen number
*D*_0_	characteristic inverse Knudsen number
*D_(x,y)_*, *D_(ε,θ)_*	Dimensionless damping coefficients
*E*_0_	perturbation amplitude of eccentricity
*H*	dimensionless film thickness, *H* = *h*/*c*
*H*_0_	Dimensionless steady film thickness
*H_E_*, *H_Θ_*	derivative of *H*_*d*0_ to. *E*_0_ and *Θ*_0_
*Kn*	Knudsen number
*K_(x,y)_*, *K_(ε,θ)_*	Dimensionless stiffness coefficients
*L*	bearing length/mm
*n*	Journal rotating speed/r·min^−1^
*N*	Dimensionless friction work consumption
*m*	molecular mass of the gas
*p_a_*	atmospheric pressure/*Pa*
*P*	dimensionless pressure, *P* = *p*/*p_a_*
*P*_0_	Dimensionless steady film pressure
*P_E_*, *P_Θ_*	derivative of *P*_*d*0_ to. *E*_0_ and *Θ*_0_
*Qcon*	flow rate for continuum flow
*Q_p_*	flow rate coefficients for Poiseuille flows
${\bar{Q}}_{p}$	dimensionless flow rate coefficients for Poiseuille flows
*r*	bearing radius/mm
*r_j_*	journal radius/mm
*R*	gas constant
*Rg*	gas constant/J·mol^−1^·K^−1^·kg^−1^
*T_a_*	temperature/K
*ε*	eccentricity ratio
*ε*_0_	eccentricity ratio in static position
*θ*	attitude angle/deg
*θ*_0_	attitude angle in static position/deg
*Θ*_0_	perturbation amplitude of attitude angle/deg
*Λ*	bearing number
*μ*	air viscosity/Pa·s
*τ*	dimensionless time
*φ*, *ψ*	cylinder coordinates
*Ω*	Perturbation frequency ratio
